# Prevalence and features of eosinophilic gastrointestinal disorders using the large real-world TriNetX database

**DOI:** 10.1016/j.jacig.2026.100762

**Published:** 2026-07-17

**Authors:** Cora E. Miracle, Noam Zevit, Edward Gerster, Kara Kliewer, Marc E. Rothenberg

**Affiliations:** a Division of Allergy and Immunology, Cincinnati Children’s Hospital Medical Center, University of Cincinnati College of Medicine, Cincinnati, Ohio; b Schneider Children’s Medical Center of Israel, Petach Tikva, Israel; c Faculty of Medicine, Tel Aviv University, Tel Aviv, Israel

**Keywords:** Eosinophilic esophagitis, eosinophilic gastritis, eosinophilic colitis, prevalence, TriNetX

## Abstract

**Background:**

There are substantial questions concerning the epidemiology and demographics of eosinophilic gastrointestinal disorders (EGID). We queried TriNetX, a large electronic medical record database, to assess EGID demographics.

**Objectives:**

Our objective for this study was to characterize the prevalence, demographic features, disease overlap, and treatment patterns of patients with EGID using a large multicenter electronic health record database.

**Methods:**

TriNetX, a deidentified patient database derived from 92 health care organizations and a total population of 129,260,452 patients, was queried for EGID. Cases were identified by ICD codes (eosinophilic esophagitis [EoE] K20, eosinophilic gastritis/enteritis [EoG/EoN] K52.81, eosinophilic colitis [EoC] K52.82) and endoscopy occurrence. Demographic and clinical characteristics were analyzed.

**Results:**

We identified 77,726 EoE, 6,554 EoG/EoN, and 2,898 EoC cases. Prevalence was approximately 1:1,673, 1:19,842, and 1:44,873, respectively. Multiple EGID occurred in 5% of all patients but in 47% of those with EoG/EoN. EGID was most common in non-Hispanic White individuals (79%). Male predominance was observed in EoE (59%) but not EoG/EoN (46%) or EoC (45%). Mean patient ages at query were 39, 31, and 40 years for EoE, EoG/EoN, and EoC, respectively (*P* < .0001). The most prescribed medications were proton pump inhibitors for EoE, antiemetics and corticosteroids for EoG/EoN, and corticosteroids for EoC.

**Conclusions:**

We identified the largest reported population of patients with EGID (77,726 cases); our findings substantiate their rarity (0.002-0.5% prevalence), male predominance limited to EoE, substantial co-occurrence of multiple EGID, and distinct age distribution of EoG/EoN. The identified cohort and approaches provide opportunities for further research.

Eosinophilic gastrointestinal disorders (EGID) are chronic inflammatory diseases characterized by the infiltration of eosinophils into various layers and regions of the gastrointestinal (GI) tract and organ-specific dysfunction.^1^ They are subdivided into eosinophilic esophagitis (EoE), eosinophilic gastritis (EoG), eosinophilic duodenitis, eosinophilic enteritis (EoN), and eosinophilic colitis (EoC).^2^ Symptoms of EGID are not uniform and depend both on the location of involvement in the GI tract and the layer of the bowel wall that is involved. For example, EoG and EoN may present with symptoms such as abdominal pain, chest pain, nausea, and vomiting.^3^ In contrast, EoC often presents with abdominal pain, diarrhea, malabsorption, and weight loss.^4^

The diagnosis of all EGID necessitates both symptoms associated with dysfunction of the involved GI segment and an eosinophil-rich infiltrate confirmed by biopsy of the affected area during endoscopic procedures (except for the extremely rare muscular and mucosal variants, which may be diagnosed during surgical procedures). Alternative conditions that may lead to eosinophilic inflammation must also be ruled out before a diagnosis is made. Such causes include but are not limited to hypereosinophilic syndrome, inflammatory bowel disease, infection, or an adverse drug reaction (eg, to tacrolimus).^5^

A global metanalysis including 40 studies with over 288 million patients from 15 countries reported EoE prevalence to be 5.31/100,000 cases.^6^ Two large studies by Jensen and colleagues, utilizing IMS LifeLink data from 75 million patients, examined the prevalence of various EGID. These studies found prevalence of EoE at 56.7/100,000 cases, meaning that EoE is no longer considered an orphan disease (<200,000 patients in the United States). Eosinophilic gastroenteritis (EGE, a former disease definition now replaced by EoG/EoN) has a prevalence of 8.4/100,000 cases and EoC 3.3/100,000 cases.^7^^,^^8^ Accurate prevalence data provide the foundation for elucidating demographic, environmental, and genetic contributors to disease development while also revealing disparities in diagnosis and access to care. Moreover, heightened awareness informed by robust epidemiologic evidence can facilitate earlier recognition by clinicians, empower affected patients, and guide public health and research priorities. Ultimately, clarifying the burden of EGID is essential for improving outcomes and advancing targeted prevention and treatment strategies.

Among the major databases used in EGID epidemiologic studies, TriNetX stands out for its unique features.^7^, ^8^, ^9^, ^10^ Unlike IMS Health LifeLink, which relies on insurance claims and prescription data, TriNetX aggregates multinational data directly from electronic health records (EHRs). This includes rich clinical details, such as procedures, laboratory results, vital signs, and medication histories, from 250 million deidentified patient health records from 92 different health care organizations. Though IBM Explorys also utilizes EHRs and incorporates some claims data, its scope is limited to the United States. A key advantage of TriNetX is its real-time data updates, enabling dynamic analyses and up-to-date insights that are not possible with static, claims-based datasets.

In this study, we extend the existing literature on the prevalence and epidemiology of EGID by leveraging the TriNetX platform. By adding information from additional health care organizations and a broader patient population, our analysis complements prior studies that are based on other large databases. Including multinational EHR data may provide further context for understanding EGID prevalence and patterns of care. These findings support ongoing efforts to improve clinical recognition, inform resource planning, and guide future research in EGID.

## Methods

The TriNetX database, using natural language processing derived from health care organizations, was accessed in July 2024 from the University of Cincinnati access network. The network with natural language processing had a total of 129,260,452 patients from 92 health care organizations at the time of the queries. These patients are mainly derived from the United States but include patients from multiple countries representing North America, Europe, the Middle East, the Asia-Pacific, Latin America, and Africa. Some of the countries within the TriNetX database include: Belgium, Bulgaria, Estonia, France, Georgia, Germany, Italy, Lithuania, Poland, United Arab Emirates, United Kingdom, Israel, Spain, Australia, India, Japan, Malaysia, Singapore, South Korea, Taiwan, Brazil, Colombia, and Ghana. Data queried extended from January 2012 to July 2024. International Classification of Disease (ICD)-10 codes for EGID and their respective diagnostic procedure were used (EoE K20, EoG/EoN K52.81, EoC K52.82) (endoscopy procedure of the esophagus Current Procedural Terminology [CPT] 1007241, flexible colonoscopy CPT 1022231). Inclusion criteria were the occurrence of at least one ICD-10 code for EGID and an associated GI diagnostic procedure in their health records (upper endoscopy for EoE or EoG/EoN, colonoscopy for EoC). Sex, age, ethnicity, race, and medication frequency were reported. The 12-year prevalence was calculated by taking the total number of patients with the specific EGID and dividing by the total number of patients within the TriNetX database. Means and 95% confidence intervals are reported. The deidentified data from TriNetX is compliant with the Health Insurance Portability and Accountability Act (certified ISO 270001:2013).^1^

## Results

**Fig 1:**
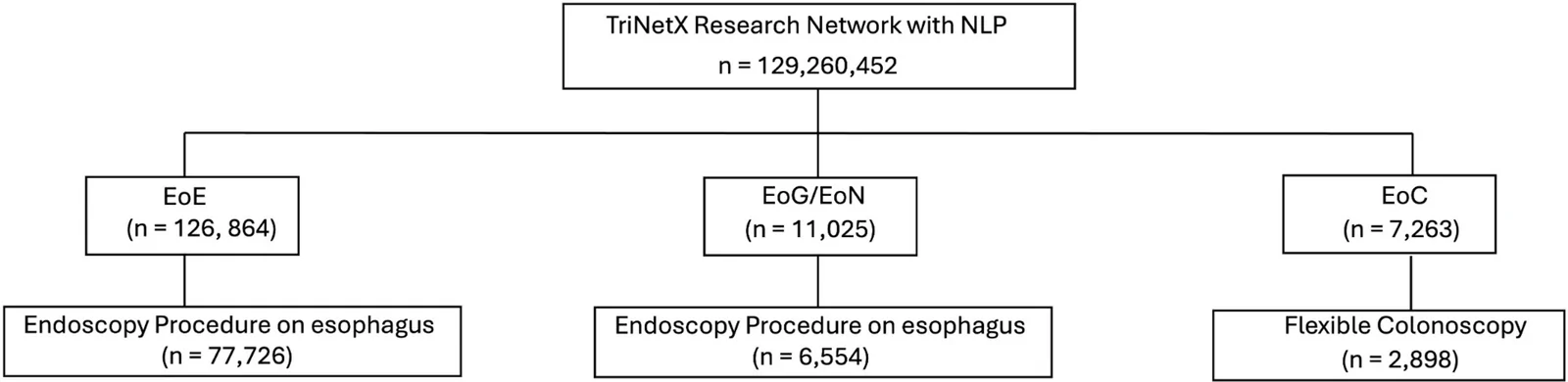
Numbers of patients with EGID queried from TriNetX database. Data queried extended from January 2012 to July 2024. ICD-10 codes for EGID and their respective diagnostic procedure were used (EoE K20, EoG/EoN K52.81, EoC K52.82) (endoscopy procedure of esophagus CPT 1007241, flexible colonoscopy CPT 1022231). Inclusion criteria were occurrence of at least one ICD-10 code for EGID and associated GI diagnostic procedure in their health records (upper endoscopy for EoE or EoG/EoN, colonoscopy for EoC). *NLP,* Natural language processing.

The TriNetX database, comprising information from 129,210,452 individuals, was queried for EGID. Using the criteria of ICD-10 coding, 126,864, 11,025, and 7,263 patients with EoE, EoG/EoN, and EoC were identified, respectively (Fig 1). Following the additional criteria of endoscopy, 77,726, 6,554, and 2,898 patients with EoE, EoG/EoN, and EoC were included, respectively.

### Demographics of TriNetX EGID population

EoE predominantly affected male subjects (59.37%) and was more common among White and non-Hispanic individuals (77.27% and 79.27%, respectively; TriNetX total population was 61% White). The average age of patients with EoE was 39 years. Similarly, EoG and EoN were most prevalent among White and non-Hispanic individuals (73.25% and 81.57%, respectively). The average age of the population with EoG/EoN was 31 years. In contrast to EoE, there was a modest trend for increased female preponderance in non-EoE EGID (Table I). The average age for patients with EoC was 40 years, with a predominance of White (72.3%) and non-Hispanic (76.07%) individuals (Table I).

**Table I:** Demographic characteristics of patients with EGID from TriNetX database

Characteristic	EoE	EoG/EoN	EoC
Age (years), mean (range; SD)	39 (0-90; 21)	31 (0-90; 21)	40 (0-90; 24)
Sex (%)			
Male	59.4	45.8	45.4
Female	40.6	53.1	52.9
Race (%)			
White	77.3	73.3	72.3
Black	6.73	10.5	8.86
Asian	1.81	2.94	3.17
Native Hawaiian	0.13	0.21	0.651
America Indian	0.24	0.43	0.357
Other	3.28	4.26	4.14
Unknown	10.5	8.39	10.6
Ethnicity (%)			
Hispanic	6.06	8.41	9.45
Non-Hispanic	79.3	81.6	76.1
Unknown	14.7	10.0	14.5

Sex, race, and ethnicity are self-reported by patient to health care organization.

### Prevalence of EGID in TriNetX

The prevalences of EoE, EoG/EoN, and EoC were 1:1,672, 1:19,839, and 1:44,872, respectively (Table II).

**Table II:** Prevalence of EGID within TriNetX database

EGID	Total no. of patients with disease	Percentage population	Prevalence	95% confidence interval
EoE	77,726	0.059	1:1,672	0.000584, 0.000611
EoG/EoN	6,554	0.005	1:19,839	0.0000492, 0.0000516
EoC	2,898	0.002	1:44,872	0.0000215, 0.0000231

Sample included 129,260,452 subjects.

### Age distribution of EGID in TriNetX

Each EGID was divided into age group cohorts: 0-6, 7-12, 13-18, 19-30, 31-50, 51-74, and ≥75 years. The TriNetX total population was mainly adults, although there was a wide age range, with 15% children (<18 years of age) (see Fig E1 in the Online Repository available at www.jaci-global.org). In EoE, the highest prevalence was observed in the 31-50 age range (29% of TriNetX network). EoG/EoN was most prevalent in early adulthood (19-30 years; 32%). In contrast, EoC showed the highest prevalence in the older population, particularly among those aged 51-74 (26%). Diagnosis of these conditions in children aged 0-6 was rare, with less than 5% prevalence across all EGID (Table III).

**Table III:** Prevalence of EGID by age group

EGID	Age range (years) (no. of subjects)	Prevalence (%)	Ratio
EoE (n = 77,726)	0-6 (2,284)	3.17	1:32
	7-12 (5,200)	7.22	1:14
	13-18 (8,469)	11.8	1:9
	19-30 (13,893)	19.3	1:5
	31-50 (20,709)	28.8	1:3
	51-74 (17,456)	24.2	1:4
	≥75 (4,003)	5.6	1:18
EoG/EoN (n = 6,554)	0-6 (236)	4	1:25
	7-12 (618)	10	1:9
	13-18 (1,127)	18.3	1:4
	19-30 (1,977)	32	1:2
	31-50 (920)	14.9	1:6
	51-74 (979)	15.9	1:5
	≥75 (289)	4.7	1:20
EoC (n = 2,898)	0-6 (61)	2.1	1:44
	7-12 (230)	8.3	1:11
	13-18 (421)	15.2	1:6
	19-30 (625)	22.6	1:3
	31-50 (423)	15.2	1:6
	51-74 (711)	25.7	1:3
	≥75 (296)	10.7	1:8

Number of cases in specific age group was divided by total number of patients for each EGID to obtain prevalence and ratio.

### Multisegment EGID in TriNetX

**Fig 2:**
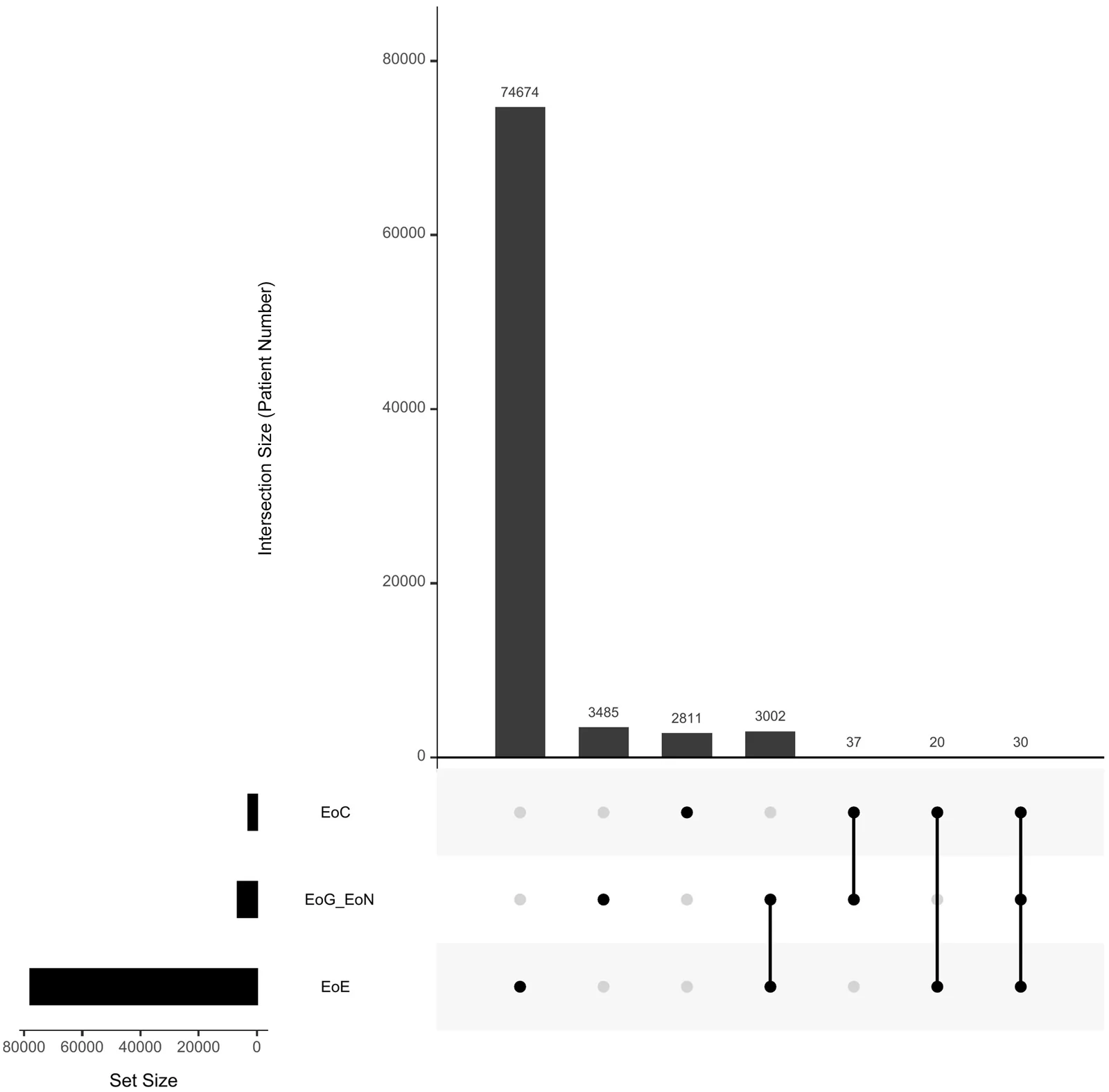
EGID co-occurrence of multisegment EGID. Upset plot depicting patient counts of various combinations of co-occurrence for EoE, EoG/EoN, and EoC from patients with EGID queried from TriNetX database (same dataset as Fig 1). EGID co-occurrence is termed *multisegment EGID.*

The occurrence of multiple EGID in the same individual, referred to as multisegment EGID, was assessed (Fig 2). Of individuals with EoE, EoG/EoN, and EoC, 4%, 47%, and 5%, respectively, had multisegment EGID. The most common overlap was between EoE and EoG/EoN. Of 6,554 patients with EoG/EoN, 3,032 also had EoE. Of the patients with EoC (n = 2,989), 50 had coexisting EoE and 67 had coexisting EoG/EoN. Of all subjects with EGID, 0.03% had all 3 EGID.

### Treatment of EGID

The most prescribed medications for patients with EGID were assessed (Table IV). Proton pump inhibitors (PPIs) were prescribed to 78% of patients with EoE, with omeprazole being the most frequently prescribed PPI (69%). For EoG and EoN, the most prescribed medications were corticosteroids and ondansetron, prescribed to 72% of patients.

**Table IV:** Most commonly prescribed medications for EGID

Medication	EoE	EoG/EoN	EoC
PPI	78	61	55
Omeprazole	69	40	34
Pantoprazole	44	28	30
Esomeprazole	20	17	14
Simethicone	19	19	21
Lansoprazole	16	14	11
Corticosteroids	67	72	77
Dexamethasone	39	43	54
Fluticasone	40	32	30
Triamcinolone	22	23	25
Budesonide	22	28	30
Ondansetron	65	72	73
Antihistamine	57	61	64
Acetaminophen	56	63	68
Bronchodilator	50	51	68
Laxative	48	49	63
Sodium bicarbonate antacid	31	31	43
Phenylephrine	15	14	19
Total	77,726	6,554	2,898

Data are shown as percentages. Medications prescribed to patients with ICD-10 code for EoE, EoG/EoN, or EoC and for endoscopic procedure. Percentage of medications prescribed was reported through TriNetX database.

## Discussion

This study is the first to examine the epidemiology of EGID using the TriNetX database. Leveraging this large multi-institutional dataset, we report that (1) EoE is rare relative to other allergic conditions (0.06% prevalence) and non-EoE EGID are rare conditions (0.002-0.5% prevalence), (2) sex predominance is limited to EoE (male predominance), and (3) there is substantial occurrence of multisegment EGID.

A 2013 study by Dellon et al, which included 80 health plans representing 78 million patients, reported the prevalence of EoE as 56.7/100,000 cases.^8^ In our study, which included 129,260,452 patients, we observed a prevalence of 59.7/100,000 cases, corroborating the findings of previous studies using EHR databases. In 2016, Jensen et al utilized the IMS Health LifeLink PharMetrics Plus Claims database to report the prevalence of EGE (now replaced by EoG/EoN) and EoC in 78 million patients.^7^ They reported EGE prevalence at 8.4/100,000 cases; since then, EGID nomenclature has changed to EoG and EoN, replacing the previously used term EGE. As a result of ICD-10 code constraints, our study assessed the prevalence of EoG/EoN, which was 5.04/100,000 cases, instead of individually assessing EoG prevalence and EoN prevalence. The prevalence of EoC in their study was approximately 3.3/100,000 cases, compared with 2.2/100,000 cases in our analysis. Overall, these findings demonstrate that population-level prevalence estimates of EGIDs remain broadly consistent across large administrative and EHR-based datasets.

Demographics in our study are consistent with other large EGID cohort studies, showing a male predominance in EoE and no sex predominance in EoG/EoN or EoC.^7^^,^^8^ A smaller study of 1,820 patients with EoG/EoN reported a female predominance.^10^ This variation is likely due to the size discrepancies. Similarly, though larger studies report no sex differences for EoC prevalence,^11^ a smaller Italian study (73 patients) showed a female predominance.^12^ Taken together, these discrepancies suggest that observed sex distributions in non-EoE EGIDs may be highly sensitive to sample size and cohort composition, reinforcing the need for larger, more representative datasets to clarify true underlying epidemiologic patterns.

We also examined EGID co-occurrence. To date, only one study, with 373 patients, reported on co-occurrence, finding that up to 41% of patients had eosinophilic infiltration outside the primary disease site.^13^ In contrast, our study found a lower co-occurrence rate, likely reflecting the larger sample size and reliance on ICD-10 codes rather than biopsy results. Though the true co-occurrence rate may be higher than reported in our study, the diagnosis of EGID using ICD-10 criteria indicates lower co-occurrence rates overall. Notably, EoE and EoG/EoN co-occur more frequently than EoE and EoC or than EoG/EoN and EoC.

Examining GI-targeted medication use provides insights into standard care and potential cost implications of newer treatments. PPIs were the most frequently prescribed medication for EoE, consistent with the report by Dellon et al.^8^ Dupilumab, approved by the US Food and Drug Administration for EoE in May 2022, was rarely prescribed by July 2024, likely due to its novelty, injectable route, and significant cost as well as the high efficacy of topical steroids. Corticosteroids were the most frequently prescribed medications for EoG, EoN, and EoC, with dexamethasone being the most commonly prescribed drug, followed by fluticasone, budesonide, and triamcinolone. Although case series are limited, systemic and GI-targeted steroids remain the mainstay of treatment for these disorders.^1^^,^^2^^,^^13^ Dietary treatments, including elimination diets, and medication administration routes could not be assessed in TriNetX.

The vast body of data in TriNetX makes it an extremely valuable resource; however, it does have several limitations. Demographic information within TriNetX is self-reported, leading to missing or unknown information on sex, race, ethnicity, and age in some cases. Additionally, age is captured as a single time point corresponding to when data were extracted from an individual institution rather than as a longitudinal variable. Because TriNetX data are derived primarily from large health care systems, the patient population may not be fully representative of all age groups, which limits interpretation of age-specific prevalence estimates. The inability to distinguish between certain EGID subtypes is another limitation. For example, the ICD-10 code for EoG also encompasses EoN. Only recently was nomenclature for EGID standardized.^2^ Cases that have been diagnosed earlier than these guidelines would have similarly pooled EoG and/or EoN together as EGE. Discrimination between these two distinct EGID was made impossible without individually assessing patient-specific data. Thus, at this point, it remains logical to assess the prevalence of both disorders together. Future studies may analyze the two for further granularity once the new nomenclature is readily used and future iterations of ICD divide the two conditions. A third limitation is that biopsy results with eosinophil counts were unavailable to ascertain diagnoses, mandating reliance on the correct diagnosis and the ICD-10 codes as well as diagnostic procedures. Though necessitating a coded diagnostic procedure along with the ICD-10 code for EGID increased the certainty of the diagnosis, patients undergoing endoscopy in private settings may not have had their procedural code in TriNetX despite being correctly diagnosed with EGID. In addition, patients may have been misdiagnosed with inflammatory bowel disease or hypereosinophilic syndrome rather than an EGID. These limitations could have led to underestimation of the number of EGID cases—more so in adults than children, who less frequently undergo endoscopy in private settings. The concordance of our findings with those of studies utilizing billing codes suggests that the extent of this bias was limited. The ICD codes that we used were K20, K52.81, and K52.82.

In conclusion, this study leveraged the TriNetX database to provide a comprehensive analysis of EGID epidemiology, including prevalence, age-specific rates, demographics, co-occurrence, and commonly prescribed medications. The prevalence of EoE was 59.7/100,000 cases, like earlier estimates. We also observed that the prevalence of EoC and EoG/EoN (formerly EGE) was modestly lower than previously reported. We found a strong male predominance in EoE and no sex difference in EoG, EoN, or EoC. Regarding co-occurrence of EGID (ie, multisegment EGID), our study found lower rates than earlier research, likely because of methodologic differences, with EoE and EoG/EoN co-occurring more frequently than other combinations. The most prescribed medications for EGID were PPIs for EoE and corticosteroids for EoG, EoN, and EoC. Despite the recent approval of dupilumab, its use is not yet widespread. By leveraging one of the largest multi-institutional datasets to date, this study provides a comprehensive and contemporary analysis of EGID epidemiology, co-occurrence, and treatment patterns, offering new and evolving insights into clinical trends and gaps that can inform both clinical care and future research.

## Disclosure statement

Disclosure of potential conflict of interest: M. E. Rothenberg is consultant for Pulm One, Spoon Guru, ClostraBio, Serpin Pharm, Celldex, Uniquity Bio, EnZen Therapeutics, Bristol Myers Squibb, AstraZeneca, Pfizer, Glaxo Smith Kline, Regeneron/Sanofi, and Guidepoint; holds equity interest in the first 7 listed and Santa Ana Bio; receives royalties from reslizumab (Teva Pharmaceuticals), PEESSv2 (Mapi Research Trust), and UpToDate; and is an inventor of patents owned by Cincinnati Children’s Hospital Medical Center. N. Zevit is consultant or has served on advisory boards of Dr Falk Pharma, Adare Pharmaceuticals, Regeneron/Sanofi, AstraZeneca, Rafa, and Takeda. The rest of the authors declare that they have no relevant conflicts of interest.

## Supplementary data

Supplementary Fig E1

## References

[1] Marasco G., Visaggi P., Vassallo M., Fiocca M., Cremon C., Barbaro M.R. (2023). Current and novel therapies for eosinophilic gastrointestinal diseases. Int J Mol Sci.

[2] Dellon E.S., Gonsalves N., Abonia J.P., Alexander J.A., Arva N.C., Atkins D. (2022). International consensus recommendations for eosinophilic gastrointestinal disease nomenclature. Clin Gastroenterol Hepatol.

[3] Chen P.H., Anderson L., Zhang K., Weiss G.A. (2021). Eosinophilic gastritis/gastroenteritis. Curr Gastroenterol Rep.

[4] Impellizzeri G., Marasco G., Eusebi L.H., Salfi N., Bazzoli F., Zagari R.M. (2019). Eosinophilic colitis: a clinical review. Dig Liver Dis.

[5] Papadopoulou A., Amil-Dias J., Auth M.K., Chehade M., Collins M.H., Gupta S.K. (2024). Joint ESPGHAN/NASPGHAN guidelines on childhood eosinophilic gastrointestinal disorders beyond eosinophilic esophagitis. J Pediatr Gastroenterol Nutr.

[6] Hahn J.W., Lee K., Shin J.I., Cho S.H., Turner S., Shin J.U. (2023). Global incidence and prevalence of eosinophilic esophagitis, 1976-2022: a systematic review and meta-analysis. Clin Gastroenterol Hepatol.

[7] Jensen E.T., Martin C.F., Kappelman M.D., Dellon E.S. (2016). Prevalence of eosinophilic gastritis, gastroenteritis, and colitis: estimates from a national administrative database. J Pediatr Gastroenterol Nutr.

[8] Dellon E.S., Jensen E.T., Martin C.F., Shaheen N.J., Kappelman M.D. (2014). Prevalence of eosinophilic esophagitis in the United States. Clin Gastroenterol Hepatol.

[9] Mansoor E., Cooper G.S. (2016). The 2010-2015 prevalence of eosinophilic esophagitis in the USA: a population-based study. Dig Dis Sci.

[10] Mansoor E., Saleh M.A., Cooper G.S. (2017). Prevalence of eosinophilic gastroenteritis and colitis in a population-based study, from 2012 to 2017. Clin Gastroenterol Hepatol.

[11] DiTommaso L.A., Rosenberg C.E., Eby M.D., Tasco A., Collins M.H., Lyles J.L. (2019). Prevalence of eosinophilic colitis and the diagnoses associated with colonic eosinophilia. J Allergy Clin Immunol.

[12] Rossi C.M., Lenti M.V., Merli S., Lo Bello A., Mauro A., Anderloni A. (2024). Clinical and atopic features of patients with primary eosinophilic colitis: an Italian multicentre study. Intern Emerg Med.

[13] Pesek R.D., Reed C.C., Muir A.B., Fulkerson P.C., Menard-Katcher C., Falk G.W. (2019). Increasing rates of diagnosis, substantial co-occurrence, and variable treatment patterns of eosinophilic gastritis, gastroenteritis, and colitis based on 10-year data across a multicenter consortium. Am J Gastroenterol.

