# Supplementary material for: Prevalence and features of eosinophilic gastrointestinal disorders using the large real-world TriNetX database
Source: J Allergy Clin Immunol Glob. 2026 Jul 17;5(5):100762. doi: 10.1016/j.jacig.2026.100762 (PMC13499153; doi:10.1016/j.jacig.2026.100762)
Supplement: Supplementary file 1 [file mmc1.docx]

**Supplementary Materials**

**Supplementary Figure 1. Age distribution of patients in TriNetX database.** TriNetX has a total of 129,260,452 patients from 92 healthcare organizations at the time of the queries, including 6,628,729 patients over the age of 90 years.
